# Question-driven summarization of answers to consumer health questions

**DOI:** 10.1038/s41597-020-00667-z

**Published:** 2020-10-02

**Authors:** Max Savery, Asma Ben Abacha, Soumya Gayen, Dina Demner-Fushman

**Affiliations:** Lister Hill National Center for Biomedical Communications, U.S. National Library of Medicine, National Institutes of Health, Bethesda, MD USA

**Keywords:** Scientific data, Computer science, Health care

## Abstract

Automatic summarization of natural language is a widely studied area in computer science, one that is broadly applicable to anyone who needs to understand large quantities of information. In the medical domain, automatic summarization has the potential to make health information more accessible to people without medical expertise. However, to evaluate the quality of summaries generated by summarization algorithms, researchers first require gold standard, human generated summaries. Unfortunately there is no available data for the purpose of assessing summaries that help consumers of health information answer their questions. To address this issue, we present the MEDIQA-Answer Summarization dataset, the first dataset designed for question-driven, consumer-focused summarization. It contains 156 health questions asked by consumers, answers to these questions, and manually generated summaries of these answers. The dataset’s unique structure allows it to be used for at least eight different types of summarization evaluations. We also benchmark the performance of baseline and state-of-the-art deep learning approaches on the dataset, demonstrating how it can be used to evaluate automatically generated summaries.

## Abstract (summary)

**Table:** 

Measurement(s)	answers to health questions • Summary
Technology Type(s)	Search Engine • human experts • computational modeling technique
Factor Type(s)	medical answers to health questions

Machine-accessible metadata file describing the reported data: 10.6084/m9.figshare.12676655

## Background & Summary

A summary is a concise description that captures the salient details from a more complex source of information^1^. Summaries are regularly used as a tool to quickly understand content from a single source, such as a book or movie, or from many disparate sources, such as news stories about a recent event. Even this article began with a summary: an abstract.

Summarization can be particularly useful for helping people easily understand online heath information. One of the first places people turn to for answers to their health questions is the internet^2^. A conventional search engine will return a set of web pages in response to a user’s query, but without considerable medical knowledge the consumer is not always able to judge the correctness and relevance of the content^3^. In fact, finding relevant biomedical material can be difficult for even medical experts^1^. While having a reliable, easy-to-understand summary for an article—such as one similar to the plain language summaries created by the health organization Cochrane^4^—would likely make searching for health information easier, it is not possible to tailor a manually generated summary to every user. For this reason, a summary automatically generated in response to a user’s query could be extremely beneficial, especially for users who do not have medical expertise.

Recent developments in automatic text summarization, a field at the intersection of machine learning and natural language processing (NLP), have shown the potential to aid consumers in understanding health information^5^. However, to develop more advanced summarization algorithms capable of reliably summarizing medical text, researchers require human curated datasets that can be used to consistently measure the quality of machine generated summaries. Unfortunately, there is currently a lack of question-driven and consumer-focused data available, i.e., human generated summaries of information relevant to helping consumers answer their health questions. A dataset for this purpose must contain the following data: (1) questions asked by people without medical expertise; (2) documents containing answers to the questions; and (3) easily understood summaries that are informed by the health questions asked by consumers. In order to address the absence of data that meets these conditions, the contribution of this paper is as follows: A new gold standard dataset, MEDIQA-Answer Summarization (MEDIQA-AnS)^6^, consisting of 156 health questions asked by consumers, corresponding answers to these questions, and expert-created summaries of these answers.

There are many available summarization datasets, but none satisfy the conditions mentioned above. For example, popular summarization datasets include the CNN-Dailymail dataset^7^, which uses headlines as summaries of news articles, and the PubMed dataset^8^, which uses abstracts as summaries of scientific articles. These can be used for training and testing summarization systems on short, open domain text and long, scientific text. Additionally, Multi-News^9^ can be used for multi-document summarization of news articles, and BioASQ^10^ can be used for question-driven, single or multi-document summarization of biomedical text. Though the BioASQ data approaches the requirements for consumer health summarization, the questions and summaries are technical in nature.

Recently, the MEDIQA 2019 shared task^11^ introduced the MEDIQA-QA dataset for answer-ranking, encouraging medical question answering research. MEDIQA-QA is uniquely suited for the purpose of this paper: It consists of consumer health questions and passages that contain information relevant to the question. This fulfills two of our three conditions for question-driven answer summarization. We therefore used the passages in MEDIQA-QA as the primary data source for MEDIQA-AnS. To extend MEDIQA-QA for summarization, we manually generated single and multi-document summaries of the passages. We also created two versions of each summary: An extractive version, consisting of content copied-and-pasted from the passages, and an abstractive version, written from scratch, using the passages as reference. This makes at least eight different types of summarization evaluations possible, including single document or multi-document summarization, on either long or short documents, and with extractive or abstractive approaches. Researchers will be able to evaluate models in a wide variety of summarization environments, whereas many previously published datasets can only be used for one or two types of evaluations. In addition to releasing MEDIQA-AnS, we include experiments using baseline and state-of-the-art summarization approaches, focusing on the single document aspect of the task, in order to benchmark the dataset for future researchers.

## Methods

### Data creation

**Fig. 1:**
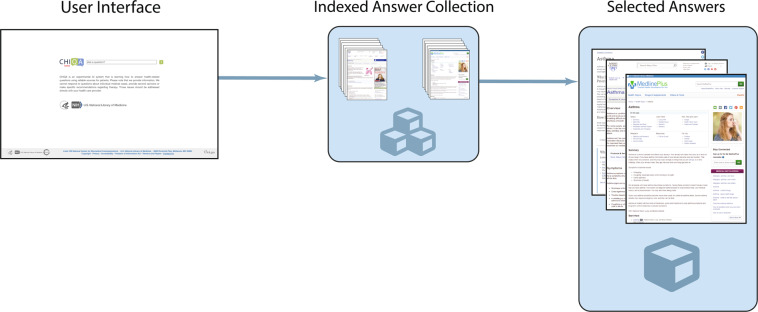
CHiQA’s user interface and answer processing pipeline.

The MEDIQA-AnS dataset introduced in this paper contains consumer health questions, the full text from reliable web pages, extracted passages from the full text, and manually created summaries. The questions in MEDIQA-AnS are a subset of those in MEDIQA-QA, consisting of questions submitted to the National Library of Medicine’s Consumer Health information Question Answering (CHiQA)^2^ online system, shown in Fig. 1. CHiQA indexes only pages hosted by reliable organizations, such as MedlinePlus and the Mayo Clinic. In response to consumers’ health questions, it provides passages from these pages, using information retrieval and machine learning techniques. The MEDIQA-QA dataset uses these passages as answers to the associated questions. It also contains manual ratings of the relevance of the passages to the question.

To create the summaries in MEDIQA-AnS, we first filtered passages from MEDIQA-QA which had been rated as “relevant but incomplete” or “excellent”. Table 1 shows the frequency of websites that this subset of answers was selected from. Then, for each question and corresponding set of answers, we generated the following types of summaries:

- Extractive summary of each answer
- Abstractive summary of each answer
- Multi-document extractive summary considering the information presented in all of the answers
- Multi-document abstractive summary

**Table 1:** Frequency of reliable websites included in MEDIQA-AnS.

Website	Frequency
medlineplus.gov	190
mayoclinic.org	151
nlm.nih.gov	44
rarediseases.info.nih.gov	39
ghr.nlm.nih.gov	31
nhlbi.nih.gov	22
niddk.nih.gov	21
ninds.nih.gov	16
womenshealth.gov	12
nihseniorhealth.gov	8
nichd.nih.gov	7
niams.nih.gov	6
cancer.gov	3
nia.nih.gov	1
nei.nih.gov	1

The summaries of the answers were generated by two subject matter experts, using a summarization interface we created to allow the annotators to input separate summaries of each type. The extractive summaries were generated by selecting chunks of text from the answers. Though the source text was sometimes punctuated correctly, it also included lists, headings, and other types of formatting. We selected text regardless of formatting, considering which chunks contained the relevant information.

For the abstractive summaries, the source text was rewritten into easier to understand, condensed sentences. Writing the abstractive summaries involved either rewording chunks, reorganizing and reformatting sentences, or potentially using extracted text that was already clear and informative. Since the answers were selected from reliable online medical resources, there were many cases in which the extractive summary was already well-worded and clear. Finally, once the extractive and abstractive summaries were written, multi-document summaries were created using all of the answers. Examples taken from the dataset can be seen in the Table 2.

**Table 2:** Examples of questions, documents, and summaries in MEDIQA-Ans.

Data type	Text
Question	*What is the consensus of medical doctors as to whether asthma can be cured?*
Document	Asthma is a condition in which your airways narrow and swell and produce extra mucus. This can make breathing difficult and trigger coughing, wheezing and shortness of breath. […]
Summary	Asthma can’t be cured, but its symptoms can be controlled. Because asthma often changes over time, it’s important that you work with your doctor to track your signs and symptoms and adjust treatment as needed […]
Question	*hi, I would like to know if there is any support for those suffering with abetalipoproteinemia? I am not diagnosed but have had many test that indicate I am suffering with this, keen to learn how to get it diagnosed and how to manage, many thanks*
Document	Bassen-Kornzweig syndrome is a rare disease passed down through families. The person is unable to fully absorb dietary fats through the intestines. Causes Bassen-Kornzweig syndrome is caused by a defect in a gene […]
Summary	Abetalipoproteimemia, also known as Bassen-Kornzweig syndrome, is diagnosed using blood tests for Apolipoprotein B, vitamin deficiencies, malformation of red blood cels, complete blood count and cholesterol. […]

### Evaluation metrics

We use ROUGE^12^ and BLEU^13^, both widely-used measures of text similarity, to calculate agreement between the annotators, compare the extractive and abstractive summaries, and evaluate the automatically generated summaries. ROUGE and BLEU measure the number of contiguous words (referred to as n-grams in NLP) occurring in a candidate summary when compared to a reference summary. For example, ROUGE-2 measures the number of contiguous two word (bigram) sequences that occur in both the candidate and reference summary, penalizing the candidate for missing bigrams. This means that ROUGE is oriented for recall, and, conversely, BLEU is oriented for precision, penalizing the candidate for including incorrect n-grams. We report ROUGE-1, ROUGE-2, ROUGE-L, and BLEU-4. Note that ROUGE-L is computed slightly differently than the other ROUGE variations, measuring the longest common subsequence between a candidate and reference.

## Data Records

We have archived nine data records with Open Science Framework (OSF), available at 10.17605/OSF.IO/FYG46^6^. The primary dataset contains 156 questions, the text of the web pages returned by CHiQA, the passages selected from the web pages, and the abstractive and extractive, multi and single document summaries of the passages. The additional eight datasets are subsets of the primary one, divided into potential experimental use cases. For example, we have included a split containing questions, corresponding web pages, and the multi-document summaries of these pages. This allows users to directly evaluate a system on multi-document summarization without having to perform additional data processing on the whole dataset. There are potentially more than eight use cases, if users are interested in using the passages as the summaries of the full text as a kind of long-form summary. However, we have not provided any pre-made splits of the data for this purpose.

**Fig. 2:**
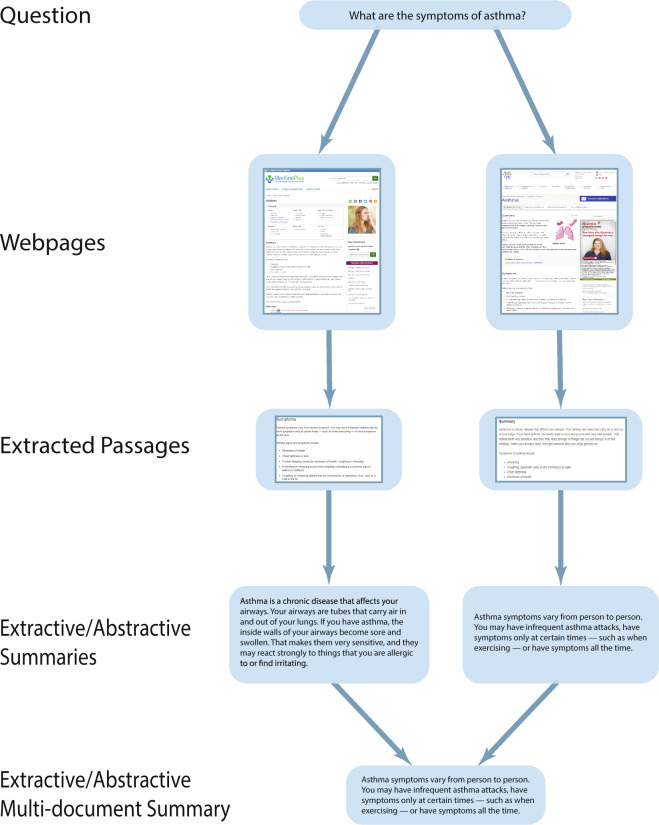
Example of a single example in the primary MEDIQA-AnS data record.

Each dataset is saved in JSON format, where each key is a question id and, depending on the dataset, each value contains a nested JSON object with the question, text of the web pages, passages, summaries, rating of passages from MEDIQA-QA, and the URL for each web page. A summary of the structure of the data for a single example is shown in Fig. 2, and statistics regarding the questions, articles, and summaries are shown in Table 3. More detailed descriptions regarding the potential use of each dataset and their respective key and value pairs can be found in the README file in the OSF archive.

**Table 3:** Average number and standard deviation of words and sentences per document type.

Data type	Count	Words		Sentences	
		Average	S.d.	Average	S.d.
Questions	156	25	31	2	2
Unique articles	348	1675	1798	95	104
Passages	552	631	869	35	48
**Summaries**					
Multi-document abstractive	156	141	119	7	6
Multi-document extractive	156	220	183	12	12
Single-document abstractive	552	83	78	4	4
Single-document extractive	552	133	127	7	8

## Technical Validation

### Inter-annotator agreement

For a subset of questions in MEDIQA-AnS, the annotators summarized the same passages, so that inter-annotator agreement between the respective summaries could be calculated. The ROUGE and BLEU scores measuring the similarity are shown in Table 4. It is apparent that the annotators more frequently use the same n-grams when creating extractive summaries. This is to be expected, as it is less likely that two individuals will use the exact same combinations of words when generating novel, abstractive text.

**Table 4:** ROUGE-2, ROUGE-L and BLEU inter-annotator agreement for each summary type.

Summarization type	ROUGE-2	ROUGE-L	BLEU
Multi-document, abstractive	0.19	0.32	0.17
Multi-document, extractive	0.56	0.57	0.49
Single document, abstractive	0.28	0.42	0.19
Single document Extractive	0.82	0.83	0.74

Additionally, we wanted to measure the similarity between the abstractive and extractive summaries. Using the abstractive summaries as the reference summary and the extracted summaries as the candidates, the scores shown in Table 5 indicate that the extractive summaries do contain many of the same n-grams as the abstractive summaries.

**Table 5:** ROUGE-2 and BLEU calculated using abstractive summaries as the reference summary and the extractive summaries as the candidate summary.

Summary type	ROUGE-2	BLEU
Single document, abstractive v. extractive	0.64	0.41
Multi-document abstractive v. extractive	0.62	0.42

**Fig. 3:**
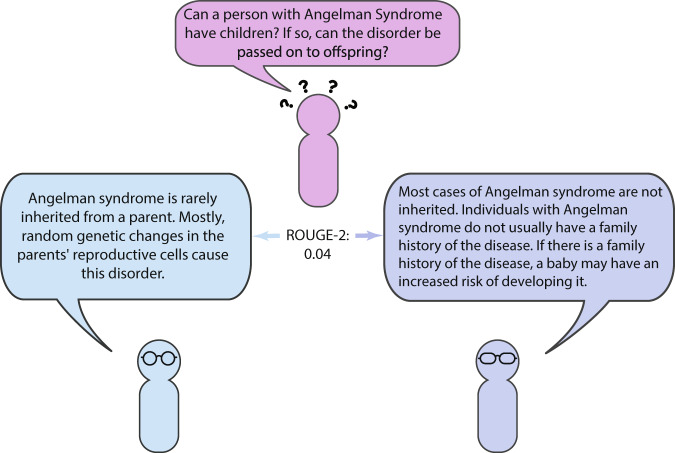
Comparison of summaries of an answer to a consumer health question. While both summaries are relevant, they receive a low ROUGE-2 score.

However, it is important to note that even if a pair of summaries receives a low ROUGE or BLEU score, neither are necessarily incorrect. To illustrate this point, Fig. 3 shows a consumer health question, two summaries written by different annotators, and the ROUGE-2 score between the summaries. While the ROUGE-2 score is quite low, both summaries contain information relevant to the question, the main difference between the two summaries being that one focuses on genetics, the other on family history. Though the metrics certainly are useful for measuring similarity, there is difficulty in quantitatively measuring the differences between summaries such as these. Fortunately, the development of metrics for this purpose is an active area of research^14^.

### Experimental benchmarking

To benchmark the MEDIQA-AnS dataset and demonstrate how it can be used to evaluate automatically generated summaries, we conducted a series of experiments using a variety of summarization approaches. Three baseline and three deep learning algorithms were implemented and are listed below:

#### Lead-3

The Lead-3 baseline takes the first three sentences of an article as a summary. This has been shown in previous work^15,16^ to be an effective baseline for summarization.

#### k random sentences

Similarly to the Lead-3 baseline, we select *k* = 3 random sentences from each article.

#### k-best ROUGE

We select *k* = 3 sentences with the highest ROUGE-L score relative to the question.

#### Bidirectional long short-term memory (BiLSTM) network

A BiLSTM^17^ was trained to select the most relevant sentences in an article, similar to other extractive LSTM models^18,19^.

#### Pointer-generator network

The Pointer-Generator network^15^ is a hybrid sequence-to-sequence attentional model, with the capability to create summaries by copying text from the source document while also generating novel text.

#### Bidirectional autoregressive transformer (BART)

BART^20^ is a recently published transformer-based encoder-decoder model combining a bidrectional encoder similar to BERT^21^ and an auto-regressive decoder similar to GPT-2^22^. To improve performance, instead of training the model directly on data relevant for summarization and other language-generation tasks, the authors first pre-trained the model on objectives designed to improve its general ability to understand the content of text. These objectives include document rotation, sentence permutation, text-infilling, token masking and token deletion. Given text which has been corrupted by one of these methods, the model is asked to de-noise the document or sequence. This pre-training procedure allows BART to generate text of higher quality when it is later fine-tuned on a more task-specific dataset, such as one for summarization. Pre-training approaches have been shown to achieve state-of-the-art results on a wide variety of NLP tasks, summarization included^20–22^.

In our experiments, all machine learning models were trained using the questions, abstracts, and snippets available in the BioASQ data, which can be easily adapted for training summarization models. Essentially, we treat the scientific abstracts in the collection as the source documents, and the snippets extracted by the creators of the collection as the summaries. The snippets provide information relevant to answering the questions, which are biomedical in domain; for example, *Is Hirschsprung disease a mendelian or a multifactorial disorder?* To compute validation loss during training, we used the medical question and answer dataset MedInfo^23^. This dataset consists of answers selected from reliable online health information, in response to consumer health questions about medications. It is therefore similar in structure and content to the MEDIQA-QA data, and can be used to approximate the single document, extractive summarization task provided in the MEDIQA-AnS collection.

We use these methods to automatically summarize the full text of the web pages in MEDIQA-AnS. Tables 6 and 7 show the comparison between the automatically generated summaries and the manually generated summaries. We include results for only single document summarization; however, the same experiments could be run in a multi-document setting.

**Table 6:** Automatically generated summaries compared to extractive summaries.

Experiment	ROUGE-1	ROUGE-2	ROUGE-L	BLEU
Lead-3	0.23	0.11	0.08	0.04
3-random	0.20	0.08	0.06	0.04
3-best ROUGE	0.16	0.08	0.06	0.00
BiLSTM	0.22	0.10	0.08	0.03
Pointer-Generator	0.21	0.09	0.07	0.03
BART	0.29	0.15	0.12	0.09

**Table 7:** Automatically generated summaries compared to abstractive summaries.

Experiment	ROUGE-1	ROUGE-2	ROUGE-L	BLEU
Lead-3	0.25	0.10	0.07	0.06
3-random	0.22	0.07	0.04	0.05
3-best ROUGE	0.17	0.07	0.04	0.02
BiLSTM	0.24	0.09	0.06	0.06
Pointer-Generator	0.24	0.08	0.06	0.05
BART	0.32	0.12	0.08	0.09

Generated examples from Lead-3, the Pointer-Generator, and BART can be seen in Table 8. These show that the quality of the source text in MEDIQA-Ans is suitable for use with generative deep learning models. The text of BART and the Pointer-Generator is grammatical and, particularly for BART, relevant to the question asked. In terms of ROUGE and BLEU, the Lead-3 baseline compares well to the other methods, as expected. Relevant to machine learning research, BART outperforms the Pointer-Generator (*p* < 0.0039, across all experiments, Wilcoxon signed-rank test), which is consistent with previous work^20^. Additionally, it is interesting to note that the ROUGE-1 and BLEU scores for each model tend to increase in the abstractive evaluation, and the ROUGE-2 and ROUGE-L scores tend to increase in the extractive evaluation. The increase in ROUGE-1 is potentially due to the fact that, as indicated in Table 3, the abstractive summaries are shorter, which makes it easier for an automatically generated summary to contain a larger percentage of unigrams present in the reference summary. ROUGE-2 and ROUGE-L may increase because it is easier for a system to get a sequence of words correct in an extractive setting. These observations may be useful to consider while using MEDIQA-AnS, since the type of summary and the metric used for evaluation will affect the observed performance of a system.

**Table 8:** Examples of summaries generated by Lead-3, Pointer-Generator, and BART, in response to consumer-health questions.

Model	Generated text
Question:	*what is copd*
Lead-3	COPD What is COPD? Chronic obstructive pulmonary disease, or COPD, is a progressive lung disease in which the airways of the lungs become damaged, making it hard to breathe.
Pointer-Generator	Overview Chronic obstructive pulmonary disease (COPD) is a chronic inflammatory lung disease that causes obstructed airflow from the lungs. Symptoms include breathing difficulty, cough, mucus (sputum) production and wheezing. It’s caused by long-term exposure to irritating gases or particulate matter, most often from cigarette smoke. People with COPD are at increased risk of developing heart disease, lung cancer and a variety of other conditions. Emphysema and chronic bronchitis are the two most common conditions that contribute to COPD.
BART	Chronic obstructive pulmonary disease, or COPD, is a progressive lung disease in which the airways of the lungs become damaged, making it hard to breathe. You may also have heard COPD called other names, like emphysema or chronic bronchitis.
Question:	*How bad can endometriosis get?*
Lead-3	Endometriosis occurs when cells from the lining of your womb (uterus) grow in other areas of your body. This can cause pain, heavy bleeding, bleeding between periods, and problems getting pregnant (infertility). Causes Every month, a woman’s ovaries produce hormones that tell the cells lining the uterus to swell and get thicker
Pointer-Generator	This growths stay in your body, and like the cells in the lining of your uterus, these growths react to the hormones from your ovaries. They grow and bleed when you get your period. Over time, the growths may add more tissue and blood.
BART	Endometriosis occurs when cells from the lining of your womb (uterus) grow in other areas of your body. This can cause pain, heavy bleeding, bleeding between periods, and problems getting pregnant (infertility). Causes Every month, a woman’s ovaries produce hormones that tell the cells lining the uterus to swell and get thicker. Your uterus sheds these cells along with blood and tissue through your vagina when you have your period.

To determine if the dataset could be used for the evaluation of question-driven summarization, we trained BART with and without access to the consumer health questions. For training and testing in these experiments, we concatenated the question to the beginning of the article. This approach is similar to other deep learning text generation work, where including unique text at the beginning of the documents fed to a model can give greater control over the content of the output. For example, users can provide the CTRL model^24^ with control codes to specify the topic of generated text.

After including the question with the text during training and evaluation, we observed a significant difference between the two types of summaries. Table 9 shows that including the question with the input documents significantly improved BART’s performance across all summarization tasks: passages and pages, extractive and abstractive (*p* < 0.0064 across all experiments, Wilcoxon signed-rank test). This indicates that being able to compare plain summaries with question-driven summaries is important to developing medical question answering systems, and since there are no existing datasets for this type of evaluation, MEDIQA-AnS can play a valuable role for further research in this area.

**Table 9:** BART using question-driven approach.

Experiment	ROUGE-1	ROUGE-2	ROUGE-L	BLEU
**Pages**				
BART + Q, Abstractive	0.32	0.12	0.08	0.09
BART, Abstractive	0.26	0.09	0.05	0.07
BART + Q, Extractive	0.29	0.15	0.12	0.09
BART, Extractive	0.24	0.10	0.07	0.05
**Passages**				
BART + Q, Abstractive	0.46	0.29	0.24	0.19
BART, Abstractive	0.43	0.27	0.21	0.17
BART + Q, Extractive	0.46	0.37	0.35	0.18
BART, Extractive	0.43	0.35	0.33	0.14

Shows summaries generated with and without access to the question, compared to extractive and abstractive summaries. Across all experiments, BART scores higher when the question (+Q) is included in the input.

## Usage Notes

We have provided instructions in the README file in the Open Science Framework repository describing how to process the MEDIQA-AnS dataset. Examples of processing the data for different summarization evaluations can be found in the code located at the GitHub repository provided below.

## Data Availability

The code to process MEDIQA-AnS and reproduce the results of the experimental benchmarks shown here can be found at https://www.github.com/saverymax/qdriven-chiqa-summarization.
